# Assessment of Correlation between Intravoxel Incoherent Motion Diffusion Weighted MR Imaging and Dynamic Contrast-Enhanced MR Imaging of Sacroiliitis with Ankylosing Spondylitis

**DOI:** 10.1155/2017/8135863

**Published:** 2017-12-27

**Authors:** Yinghua Zhao, Qun Zhang, Wei Li, Yanqiu Feng, Yihao Guo, Zhiming Xiang, Shaolin Li

**Affiliations:** ^1^Department of Radiology, Third Affiliated Hospital of Southern Medical University and Academy of Orthopedics, 183 Zhongshan Da Dao Xi, Guangdong Province, Guangzhou 510630, China; ^2^School of Biomedical Engineering and Guangdong Provincial Key Laboratory of Medical Image Processing, Southern Medical University, 1023-1063 Sha Tai Road, Guangzhou, China; ^3^Department of Radiology, Guangzhou Panyu Center Hospital, 8 Fuyu Dong Road, Panyu District, Guangzhou 511400, China; ^4^Department of Medical Imaging, The Fifth Affiliated Hospital, Sun Yat-Sen University, 52 Meihua Dong, Zhuhai 519000, China

## Abstract

The relationships between IVIM and DCE-MRI parameters in AS are not clear. We explore the correlation between intravoxel incoherent motion (IVIM) diffusion weighted imaging (DWI) and dynamic contrast-enhanced (DCE) parameters obtained on MR images in patients with ankylosing spondylitis (AS). Forty-four patients with AS were prospectively examined using a 1.5-T MR system. IVIM DWI was performed with 11 *b* values (range, 0–800 s/mm^2^) for all patients. The correlation coefficients between IVIM and DCE-MRI parameters were analyzed using Spearman's method. Our results showed that intra- and interobserver reproducibility were excellent to relatively good (ICC = 0.804–0.981; narrow width of 95% limits of agreement). Moderate positive correlations were observed between pure molecular diffusion (Ds) and maximum enhancement (ME) and relative enhancement (RE) (*r*  =  0.700, *P* < 0.001; *r* = 0.607, *P* < 0.001, resp.). Perfusion-related diffusion (Df) showed negative moderate correlation with ME (*r*  =  −0.608, *P* < 0.001). However, no correlation was observed between perfusion fraction (*f*) and any parameters of ME, RE, TTP, and BE (*r* = −0.093–0.213; *P* > 0.165). In conclusion, the IVIM parameters, especially f, might play a critical role in detecting the progression of AS, because it can provide more perfusion information compared with DCE-MRI; besides the IVIM MRI is a noninvasive method.

## 1. Introduction

The prevalence of ankylosing spondylitis (AS) is 0.1–2% of the general population. The AS is a chronic disease with active and inactive stage and it is very important to detect the active stage of AS [1]. Sacroiliitis is the most common manifestation of AS with an upward trend to involve vertebral facet joints and other auxiliary structures. Thanks to recent introductions of dynamic contrast-enhanced (DCE) magnetic resonance imaging (MRI) and Diffusion weighted imaging (DWI), AS could be diagnosed early, and the active or inactive stages of AS could be detected via observation on sacroiliitis. Moreover, previous studies have shown that DCE-MRI and DWI could predict response to the therapy by antitumour necrosis factor-*α* (TNF- *α*) in AS [2, 3].

DCE-MRI allows for estimation of tissue perfusion and permeability [3]. Specifically, using pharmacokinetic models from a DCE-MRI acquisition, semiquantitative and quantitative hemodynamic parameters, including relative enhancement (RE), maximum enhancement (ME), time to peak (TTP), and brevity of enhancement (BE), have been processed and modeled [4, 5]. Moreover, Bane et al. have reported that those parameters of the active group were significantly higher than those of the inactive group with AS and showed that DCE-MRI was valuable in assessing treatment of AS [6]. However, parameters of DCE-MRI are influenced by the injection duration time, circulation time, and contrast dosage. It should be noted that the contrast agents might increase the risk of renal fibrosis [7, 8].

DWI method enables showing the microscopic biologic structure and diffusion of water protons in tissue, without requiring an intravenous contrast agent [9]. DWI, now part of most AS MR protocols, is an effective tool for diagnosing the activity of AS and is used to monitor AS [3, 10]. A novel DWI, intravoxel incoherent motion (IVIM) is increasingly being used in AS diagnosis, with the ability to separately reveal the pure diffusion coefficient (*D*) and perfusion-related incoherent microcirculation (*f*, Df) at the same time [11]. It is based on the theory that using a series of multiple values of *b*, DWI signals attributes to perfusion in the capillary network in the low *b*-values range (*b* < 100–150 s/mm^2^), and in the high *b*-values range, the diffusion of water protons could be explored in tissue [12, 13].

Many studies have already demonstrated correlations of perfusion-related IVIM parameters and DCE-MRI perfusion pharmacokinetic parameters in brain, hepar, breast, and so on [14–17]. Some studies supported the fact that the perfusion-related IVIM parameters were significantly correlated with changes in conventional perfusion techniques in cervical cancer, squamous cell carcinoma, nasopharyngeal carcinoma, and so on [14, 18, 19]. On the contrary, other studies showed that there was no correlation between aforementioned parameters in liver cirrhosis, lung cancer, and so on [20, 21]. However, for AS, a valid theory of the relationships between IVIM and DCE-MRI parameters is still lacking. Therefore, the aim of our study was to evaluate the relationship between IVIM parameters obtained using bicomponent analysis and the perfusion obtained using DCE MR imaging in AS.

## 2. Patients and Methods

### 2.1. Patient's Selection

Our prospective study was reviewed by the ethics committee in our hospital, and the approval with written informed consent was obtained from each participant. From December 2015 to November 2016, all patients who were enrolled our study were undergoing IVIM DW imaging and DCE imaging of the sacroiliac joint. Meanwhile, they also were assayed for blood sedimentation rate (ESR), C-reactive protein (CRP), and BASDAI.

### 2.2. MR Imaging Techniques

All MR imaging was performed using a 1.5-T MR (Achieva 1.5 T, Philips Healthcare, Best, the Netherlands) and utilizing a 4-channel SENSE-body coil with high-gradient performance (amplitude 33 mT/m, slew rate 80 mT/m/ms). Four standard sequences MR imaging was performed: (A) T_1_-weighted turbo-spin-echo (TSE) with fat-saturated [echo time (TE)/repetition time (TR), 20 ms/60 ms], (B) Short tau inversion recovery (STIR) (TE/TR, 60 ms/1437 ms; echo train length, 15), (C) Scan parameters of three-dimensional volumetric interpolated fat-suppressed sequence: TE = 2.2 ms, TR = 4.7 ms, the TR is the time between each echo specific for this readout; two different flip angles (5° for pre- contrast scan, and 12° for dynamic scan); acquisition matrix, 112 × 110; FOV, 400 mm × 250 mm; slice thickness, 4 mm; number of slices, 40; SENSE factor, 0; A precontrast 3D T1 FFE scan was followed by a DCE-MRI; 0.1 mmol/kg body weight of gadopentetate dimeglumine contrast agent (Magnevist; Bayer Healthcare, Berlin, Germany) was administered intravenously at a rate of 3 ml/s, followed by a 20-ml saline flush with high-pressure injector after the acquisition of four baseline dynamic scans; each 40-slice set was collected at 25 time points for approximately 8 minutes of scanning. The spatial resolution of DCE-MRI sequence was compromised for a good temporal resolution with 10.6 s, and (D) diffusion weighted MR imaging. A total of 11 *b*-values were used: 0, 10, 20, 30, 50, 80, 100, 120, 200, 700, and 800 s/mm^2^. Axial MR images with bilateral sacroiliac joints coverage were collected (Table 1).

### 2.3. Image Analysis

#### 2.3.1. Analysis of IVIM Parameters

The DWI signal follows the biexponential model to calculate the signal attenuation IVIM, as [22]

(1) $$\begin{array}{c} \frac{S_{b}}{S_{0}}=\left(\;1\text{–}f\;\right)\exp\left(\;-bDs\;\right)+f\exp\left[\;-b\left(\;Df+Ds\;\right)\;\right], \end{array}$$

where *S*_*b*_ and *S*_0_ are the signal intensities in the pixels with different *b*-values of *b* and 0. Ds could be obtained by (2) using *b*-values greater than 200 s/mm^2^ [23], and then, using all *b*-values, the perfusion fraction of tissues (*f*) and the pseudo-diffusion coefficient (Df) were calculated by substituting Ds into (1).

(2) $$\begin{array}{c} S_{b}=S_{0}\exp-\left(\;-bDs\;\right). \end{array}$$

All curve-fitting algorithms were performed using a home developed program based on MATLAB (MathWorks, Natick, MA) and developed by the Guangdong Provincial Key Laboratory of Medical Imaging Processing, School of Biological Engineering, Southern Medical University, which has been used to perform the IVIM data of liver [24]. The parametric maps of Ds, *f*, and Df were abstracted by biexponential fitting.

#### 2.3.2. Analysis of DCE MR Parameters

For DCE MR parameters analysis, all data were transferred to a computer equipped with manufacturer-supplied software (The Netherlands Easy Vision, release 4.4) and workstation (Philips Medical Systems, Best, The Netherlands).

For DCE-MRI, ME, RE, TTP, and BE were automatically calculated by manually drawing four different regions of interest (ROIs) from time-signal intensity curves (TSIC) in our study, as yet reported by Kambadakone and Sahani [25]. ME is the difference between the maximum (SI_max_) and the precontrast signal intensity (SI_0_), which is related to the volume of the extracellular space. RE is the percentage of signal intensity increases between the SI_max_ and the SI_0_, which reflects the plasma volume (Vp) and is highly correlated with microvessel density (MVD) and vascular endothelial growth factor (VEGF). TTP reflects the time from SI_0_ to SI_max_ within a region of interest, which is directly related to permeability surface area [26]. BE is the time between wash in and wash out of contrast agents [27].

#### 2.3.3. Regions of Interest (ROI) Analysis

All regions of ROIs were positioned on DCE and DW images with *b*-values of 0 by two radiologists, each with 10 years of experience in reading musculoskeletal system MR images with the double-blind method. The specific methods used were as follows.

Bone marrow edema (BME) of sacroiliitis in conventional MR images, DW images (*b* = 0 s/mm^2^), and DCE-MRI were used as references to determine lesion areas on IVIM and DCE-MRI parametric maps. Regions of interest (ROI) were manually positioned inside each lesion at its maximum transverse level, being along approximately 1 mm inward its contour. Meanwhile, the ROI was chosen to be as large as possible with minimum contaminations from surrounding unintended tissues, such as blood vessels, necrosis, and cystic area. However, if there were no BME in sacroiliitis, the ROI was manually placed on each side of the juxta-articular bone marrow, being adjacent to bone cortical or cartilage. At last, 4 ROIs were obtained with a size of ~2 mm^2^ on IVIM and DCE-MRI of each patient, and the average of the measurement was taken.

### 2.4. Statistical Analysis

All analyses were performed using GraphPad Prism 7.03 for Windows (GraphPad Prism, USA). Normality and homogeneity of variance were, respectively, performed by the Shapiro-Wilk test and the Levene test. IVIM and MRI-DCE parameters are reported as the mean ± standard deviation of all measurements. The three sets of measurements were obtained by two radiologists (radiologist 2 had 2 sets). In order to evaluate intra- and interobserver variability, the intraclass correlation coefficient (ICC) and coefficient of variation (CV) were calculated for the three sets of measurements. Intra- and interobserver agreement was evaluated using ICC and Bland-Altman analysis. The differences among the three sets of data and their average were assessed using one-factor Analysis of Variance (one-way ANOVA). Spearman's rank correlation coefficient was calculated to measure the association between the IVIM and MRI-DCE parameters. Analyses of Spearman's correlation coefficient between IVIM and DCE-MRI parameters were performed. For all tests, two-tailed* P* values < 0.05 were considered statistically significant.

## 3. Results

### 3.1. Patients

According to criteria of European Spondyloarthropathy Study Group in 1991 [28], a total of 44 patients finally were included (32 male, 12 female; mean age, 30.11 ± 14.66 years, range, 12–59 years; duration, 5.45 ± 5.9 years; CRP, 14.23 ± 21.39 mm/L; ESR, 24.62 ± 25.11 mm/H), and 19 patients were excluded because of unconfirmed AS, spondyloarthritis (SpA), osteoarthritis (OA), rheumatoid arthritis (RA), and scoliosis.

### 3.2. DCE and IVIM Parameters

**Figure 1:**
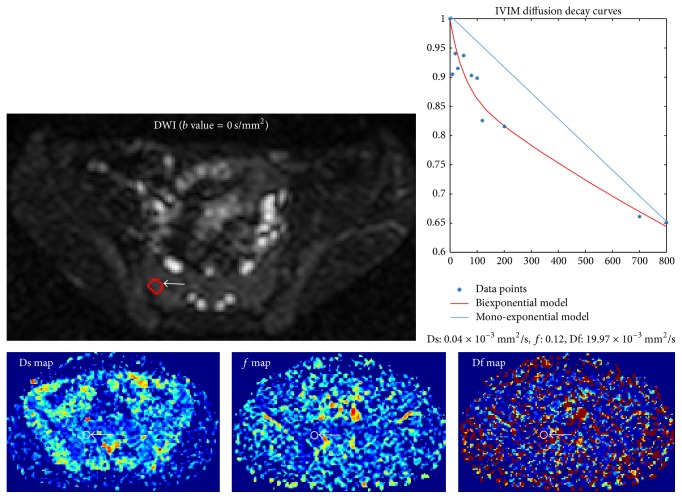
Intravoxel Incoherent Motion (IVIM) Diffusion Weighted MR imaging (DWI) in a 16-year-old male patient with duration of 3-year diagnosed ankylosing spondylitis (AS). On the Ds (pure molecular diffusion) map, *f* (perfusion fraction) map, and Df (perfusion-related diffusion) map, ROIs are placed in the juxta-articular bone marrow with sacroiliitis of AS. IVIM diffusion decay curves were shown with increasing *b* values (0, 10, 20, 30, 50, 80, 100 120, 200, 700, and 800 s/mm^2^) based on monoexponential model (blue line) and biexponential model (red line) (Ds: 0.04 ×10^−3^ mm^2^/s, *f*: 0.12, Df: 19.97 × 10^−3^ mm^2^/s). The combination of their log plots of SI versus *b*-value (the curves from top to bottom within *b* < 200 s/mm^2^) IVIM DWI signal intensity decay shows a nonlinear relation for the ROI.

**Figure 2:**
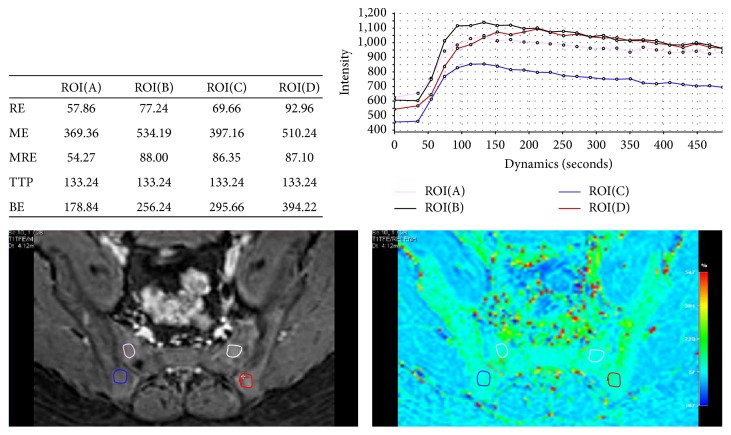
Dynamic contrast-enhanced magnetic resonance imaging (DCE-MRI) in a 16-year-old male patient with duration of 3-year diagnosed ankylosing spondylitis (AS). On the contrast-enhanced T1-weighted MR image, four ROIs were drawn on each side of the juxta-articular bone marrow with sacroiliitis. The four time-signal intensity curves (TSICs) showed four measurements of relative enhancement (RE), maximum enhancement (ME), time to peak (TTP), and brevity of enhancement (BE).

The Shapiro-Wilk test showed that all DCE (RE, ME, TTP, and BE) and IVIM (Ds, *f*, and Df) parameters had normal distributions (all *P* > 0.05), and the Levene test revealed that all the variances were homogeneous (all *P* > 0.05).  Table 2 summarizes the mean and standard deviation values of the IVIM and DCE parameters. There was no significant difference among three observations of parameters (RE, ME, TTP, BE, Ds, *f*, and Df), determined by observer 1 and 2 (*P* = 0.825–0.963).  Table 3 presented the fact that the data of intra- and interclass coefficient correlation was 95% CI, ranging from 0.804 to 0.981.  Figure 1 showed that there was a significant difference between monoexponential and biexponential curves of the IVIM diffusion in sacroiliac joints.  Figure 2 illustrates the methods used to measure the parameters in the AS study in one example of the MRI-DCE images.

### 3.3. Intra- and Interobserver Reproducibility

Table 4 revealed that measurements of the DCE (RE, ME, TTP, and BE) and IVIM (Ds, *f*, and Df) parameters were very reliable, possessing excellent intra- and interobserver reproducibility with the 95% CL ranging from 0.804 to 0.981. The coefficient of variation (CV) ranged from 2.6 to 11.07% (Table 3). Intra- and interobserver variations were found to be very low (Table 4), except for the ME value with −66.44–66.77 and −71.47–40.48%, intra-and interobserver variations, respectively.

### 3.4. Correlation between DCE and IVIM Parameters

**Figure 3:**
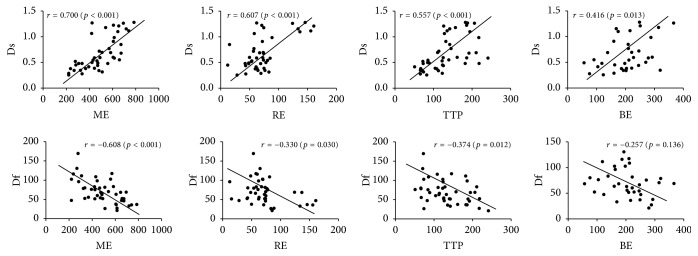
Correlation plots between pure molecular diffusion (Ds) and maximum enhancement (ME), Ds and relative enhancement (RE), Ds and time to peak (TTP), Ds and brevity of enhancement (BE), perfusion-related diffusion (Df) and ME, Df and RE, Df and TTP, and Df and BE in the juxta-articular bone marrow with sacroiliitis of ankylosing spondylitis.

The correlations between the DCE-MRI parameters (RE, ME, TTP, and BE) and IVIM (Ds, *f*, and Df) parameters were evaluated using Spearman's *R* values and shown in Table 5. Moderate and positive correlations were, respectively, discovered between Ds and some of DCE parameters, including ME (*r* = 0.700, *P* < 0.001) and RE (*r* = 0.607, *P* < 0.001). The Ds to TTP (*r* = 0.557, *P* < 0.001) and Ds to BE (*r* = 0.416, *P* = 0.013) correlations were poor. The Df to ME correlation was moderate and negative (*r* = −0.609, *P* < 0.001), while the Df to RE and TTP correlations were poor and negative (*r* = −0.330, *P* = 0.030; *r* = −0.375, *P* = 0.012, respectively). However, there were no correlations found between *f* and any parameters of ME, RE, TTP, and BE (Figure 3).

## 4. Discussion

In our study, signal-to-noise ratios (SNRs) in the raw EPI images were exceeded over 13.5, and 8 equally spaced *b*-values under 200 s/mm^2^ were performed. Previous studies show that low SNRs affect the measurement at low *b* values in IVIM [29], and that few low *b*-values under 100 s/mm^2^ (0, 50, and 100 s/mm^2^) might decrease the accuracy of IVIM measurements [15, 20]. Therefore, the reliable Df and *f* were obtained in sacroiliitis with AS.

In general, parameters of IVIM can be interpreted as separate intra- and extravascular compartments, with the parameter Ds referring to extravascular compartments, and with the parameters *f* and Df referring to the intravascular compartment [20]. Moreover, Ds, *f*, and Df are not influenced by each other [30, 31]. The perfusion parameters of IVIM pertained to different characteristics; namely, Df values reflect endovascular blood flow velocity and the mean capillary segment length, while *f* values reflect the volume of vascular and extracellular space [30]. Both of them correlate with the amount of normal angiogenesis with intact vessels in terms of basement membrane thickness and pericyte coverage [32]. They could detect the increasing number of capillaries and blood vessels involved in angiogenesis and new bone formation in sacroiliitis with AS [11, 33].

Previous studies demonstrated that DCE-MRI necessarily involves not only exchange dynamics of intra- and extravascular but also other factors, such as blood vessel density, the pattern of blood delivery, vascular permeability, and distribution of contrast agent in lesions [34]. Parameters of DCE-MRI are related to microvascular volume or flow that can reflect some composite encompassing total tracer transit. Among parameters of DCE-MRI, (1) ME is related to the volume of the extracellular space, (2) RE reflects Vp and MVD, (3) TTP is directly related to the constant rate between extravascular space and blood Vp (permeability surface area) [26], which is generally affected by the injection and circulation time [7], and (4) BE also depends on various factors, like contrast dosage, MVD, blood flow, capillary wall permeability, composition of the extracellular space, and so on [8].

We found moderate and positive correlations between Ds in IVIM and ME and RE from DCE-MRI within sacroiliitis with AS. Our results were consistent with results reported by Bourillon et al. with the correlation between Ds and ME [35]. This might be explained by considering restricted diffusion within BME in AS because of inflammatory cells increase, the increase in the volume of extracellular space and MVD associated with angioedema [10, 35]. Poor and positive correlations between Ds in IVIM, and TTP and BE from DCE-MRI also were observed in sacroiliitis with AS. It has been proved that TTP increased due to the delayed enhancement within sacroiliitis in AS [6], and it might be accompanying higher BE.

We also found a moderate and negative correlation between Df and ME, which was not consistent with the result reported by Bane et al. in renal parenchyma with the poor and negative correlation between Df and ME [6]. This different result might be caused by the reliable Df obtained in our study. The correlation between Df and ME can imply that the amount of blood and serum of capillary transported to the marrow cavity was increased [11], and the volume of the extracellular space was decreased, in sacroiliitis with AS.

Poor and negative correlations between Df in IVIM and RE, TTP, and BE from DCE-MRI were obtained. François et al. have reported that the change of edema, myxoid, fibrosis, bone sclerosis, and the number of hematopoietic and fat cells contribute to comprehending of the MRI findings in sacroiliitis with AS [36]. These histopathologies of AS could explain partially the poor and negative correlations between the Df in IVIM and RE, TTP, and BE from DCE-MRI. Other influential factors might exist, like MVD, blood flow, capillary wall permeability, composition of the extracellular space, the injection time, contrast dosage, and so on [7, 8]. Hence, this poor correlation between Df, and TTP and BE might be meaningless.

On the contrary, there are no significant correlations between *f* and the DCE-MRI parameters in our results. Zhao et al. have shown that *f* had no statistical difference between active and inactive stage of AS [11]. Conversely, Bane et al. had reported statistical differences for the parameters of DCE-MRI between the active and inactive stage of sacroiliitis [6]. Both of results support our conclusion that *f* was not correlated with the parameters based on DCE-MRI. Our results might demonstrate that IVIM provides the different perfusion information with DCE-MRI due to exogenous contrast agents and the different biophysical sensitivities, although both of them could reflect vascular volume and extracellular space [30]. Therefore, we speculated that *f* might explain the recurrence of sacroiliitis without contrast-enhancing lesion. Further studies should be performed to reveal the reason why *f* in sacroiliitis increases in the inactive stage of AS.

There were some limitations in our study. First, both DCE-MRI and DWI require longer scan times. The increased likelihood of patient movement increases susceptibility to motion artifacts and could introduce errors for the curve fit in the postprocessing phase biexponential T2^*∗*^ mapping. Movement of the subjects was minimized by careful body fixation, and the images were coregistered in postprocessing. It is difficult to accurately match ROI of IVIM and DCE-MRI in juxta-articular bone marrow with sacroiliitis of AS, and partial volume effects might result in a little error of parameters between IVIM and DCE-MRI in the lesion. Second, in our study, due to the impact of SNRs and spatial resolution, small lesions (<5 mm) in BME were easily missed. Third, because only semiquantitative parameters were derived from DCE, the correlation between perfusion fraction and any DCE parameters should be studied through quantitative parameters, like mean transit time, arterial/blood vessel fraction, total flow, and so on. Finally, some heterogeneous flow preconditions, such as the rate of injection, impaired cardiac output, and proximal arterial stenosis, were not considered in parameters of DCE-MRI, which might affect perfusion parameters of DCE-MRI in lesions with AS, while parameters from IVIM could be measured repeatedly without concerning effects of exogenous contrast agents on renal functions. However, the SNR with 1.5 T and 4 channel body coils is relatively very low. Maybe a unilateral imaging will make the SNR higher in the future. The imaging time for DWI is quite long even with 6 mm slice thickness. A better resolution with maybe fewer *b* values needs to be tested to make it more clinically useful. In the future, we will standardize MR imaging protocols and generation of parametric imaging, and we will perform model-based dynamic analytical parameters, and we will calculate mean transit time, arterial/blood vessel fraction, total flow, and so on. Whether *f* correlates with the total flow/MTT is particularly interesting and important.

Accompanying AS treatment, a long-term observation on IVIM should be carried out, exploring the role of *f* in the recurrence of active symptoms from an inactive stage of AS.

## 5. Conclusions

In this study we suggest that the IVIM perfusion imaging using 1.5-T MRI can be used in diagnosis and detection of the progression of AS. Ds in IVIM showed moderated positive correlations with ME and RE from DCE-MRI, and Df showed a moderate negative correlation with ME. However, *f* was not related to any parameters of ME, RE, TTP, and BE. The IVIM MRI has be highlighted as a novel and effective technique to detect the progression of AS, because it can provide more perfusion information compared with DCE-MRI; besides the IVIM MRI is a noninvasive method.

## Figures and Tables

**Table 1:** IVIM DW imaging and DCE MR imaging parameters.

Parameter	DCE imaging	IVIM DW imaging
Acquisition	Axial	Axial
Repetition time (ms)	4.7	3552
Echo time (ms)	2.2	61
*b* values (sec/mm^2^)		0, 10, 20, 30, 50, 80, 100 120, 200, 700, 800
Echo-planar imaging factor		3.5
Field of view (mm)	400/250	400/250
Section thickness (mm)	4	6
Intersection gap (mm)	1	1
Flip angle (°)	5, 12	90
Parallel imaging factor		2
Imaging time (min)	8	8
Number of acquisition	10	6
Acquisition matrix	112 × 110	160 × 128

**Table 2:** Distribution of IVIM and DCE-MRI parameters in sacroiliitis with AS.

Parameters	Reader 1 (*n* = 44)	Reader 2 first session (*n* = 44)	Reader 2 second session (*n* = 44)	Total (*n* = 132)	*F*	*P* ^†^
RE (%)	71.75 ± 33.58	73.69 ± 33.76	71.69 ± 31.73	72.67 ± 33.08	0.037	0.963
ME (%)	507.54 ± 144.15	487.17 ± 154.12	495.69 ± 150.91	492.85 ± 148.39	0.048	0.953
TTP (s)	138.53 ± 48.55	133.87 ± 53.45	132.69 ± 46.99	132.29 ± 48.98	0.059	0.942
BE (s)	208.24 ± 69.19	200.97 ± 80.04	207.81 ± 81.93	205.67 ± 76.58	0.092	0.912
Ds (×10^−3^ mm^2^/s)	0.69 ± 0.33	0.66 ± 0.32	0.67 ± 0.27	0.67 ± 0.31	0.043	0.957
*f*	0.15 ± 0.07	0.16 ± 0.08	0.15 ± 0.06	0.15 ± 0.07	0.193	0.825
Df (×10^−3^ mm^2^/s)	69.52 ± 29.81	71.07 ± 34.14	73.41 ± 29.27	71.33 ± 30.95	0.173	0.841

RE: relative enhancement; ME: maximum enhancement; TTP: time to peak; BE: brevity of enhancement; Ds: pure diffusion coefficient; *f*: perfusion fraction; Df: pseudo-diffusion coefficient. Data are a mean ± standard deviation. ^†^One-way ANOVA. Statistically significant (*P* < 0.05).

**Table 3:** Intra- and interobserver reproducibility in the assessment of IVIM and DCE-MRI parameters in sacroiliitis with AS.

Parameter	Intra- and interclass coefficient correlation (95% CI)		Coefficient of variation (%)	
	Intraobserver	Interobserver	Intraobserver	Interobserver
RE (%)	0.966 (0.940–0.982)	0.962 (0.932–0.979)	8.55	4.57
ME (%)	0.981 (0.966–0.989)	0.972 (0.950–0.985)	4.47	3.65
TTP (s)	0.974 (0.756–0.967)	0.935 (0.868–0.969)	8.31	6.87
BE (%)	0.831 (0.688–0.911)	0.804 (0.843–0.897)	11.07	4.62
Ds (×10^−3^ mm^2^/s)	0.956 (0.922–0.975)	0.924 (0.867–0.957)	7.23	3.78
*f*	0.979 (0.962–0.988)	0.967 (0.941–0.982)	4.8	2.63
Df (×10^−3^ mm^2^/s)	0.883 (0.805–0.937)	0.889 (0.806–0.938)	10.61	7.01

RE: relative enhancement; ME: maximum enhancement; TTP: time to peak; BE: brevity of enhancement; Ds: pure diffusion coefficient; *f*: perfusion fraction; Df: pseudo-diffusion coefficient; CI: confidence interval.

**Table 4:** Intra- and interobserver variation in the assessment of IVIM and DCE-MRI parameters in sacroiliitis with AS.

Parameters	RE (%)	ME (%)	TTP (s)	BE (s)	Ds (×10^−3^ mm^2^/s)	*f*	Df (×10^−3^ mm^2^/s)
Intraobserver	−23.73–24.94	−66.44–66.77	−30.00–52.74	−64.36–50.3	−0.36–0.24	−0.03–0.04	−39.6–28.12
Interobserver	−15.62–30.76	−71.47–40.48	−28.12–22.97	−10.31–53.98	−0.19–0.22	−0.04–0.03	−35.75–30.60

RE: relative enhancement; ME: maximum enhancement; TTP: time to peak; BE: brevity of enhancement; Ds: pure diffusion coefficient; *f*: perfusion fraction; Df: pseudo-diffusion coefficient. Values are presented as 95% limits of agreement (LoA, limits of agreement; mean interdevice difference; spans of limits of agreement).

**Table 5:** Correlations between IVIM and DCE-MRI parameters in sacroiliitis with AS.

Parameters	Ds		*f*		Df	
	*r*	*P* value	*r*	*P*-value	*r*	*P* value
ME (%)	0.700	<0.001	−0.007	0.965	−0.608	<0.001
RE (%)	0.607	<0.001	−0.093	0.553	−0.330	0.030
TTP (s)	0.557	<0.001	0.2130	0.165	−0.374	0.012
BE (s)	0.416	0.013	0.1535	0.378	−0.257	0.136

RE: relative enhancement; ME: maximum enhancement; TTP: time to peak; BE: brevity of enhancement; Ds: pure diffusion coefficient; *f*: perfusion fraction; Df: pseudo-diffusion coefficient. ^†^Statistically significant (*P* < 0.05), Spearman's rank correlation.
